# On the Treatment of Acute Rheumatism

**Published:** 1864-02

**Authors:** Thomas Inman

**Affiliations:** Physician to the Liverpool Royal Infirmary


### ON THE TREATMENT OF ACUTE RHEUMATISM.

By Dr. THOMAS INMAN, Physician to the Liverpool Royal Infirmary.

I know no fallacy in medicine upon which theories have been built more marked than that the sour smelling perspiration is evidence of the elimination of a poison, and that the poison eliminated is an acid, and consequently that alkalies are the remedies *par excellence.* The peculiar smell is simply the result of decomposition. The theory is as untenable as one founded upon the ammoniacal smell of a baby's foul napkin. Only fancy the absurdity of treating a bad typhus case with some acid because the bed linen smelt ammoniacal from the effect of incontinence of urine and the difficulty of renewing the sheets frequently! Yet on precisely similar grounds the generally received pathology of acute rheumatism has been based!

When such an untenable proposition is held by medical professors, a theory so baseless as to prevent the adherence of any one with ordinary common sense, we ought to be more charitably disposed to other theorists than we are and have been. But ignorance is always intolerant and wilr continue to be, in spite of moralists.

Since I have adopted Dr. Ree's suggestion, however, and treated my patients with lime-juice alone, the result has been far different.

During the ten years I have been a hospital physician, I have had under my care a hundred cases of acute rheumatism, and all of them have been treated with lime-juice at the rate of eight ounces per day. In five the heart has become affected, but in all, the affection has been transitory. Not one has left the hospital with a permanent cardiac disease. One patient died suddenly; he had recently had pneumonia as a complication, which passed off in two days; he was well enough to sit up in bed, and was talking vivaciously, when he suddenly died—no *post mortem* was allowed.

The average duration of the cases under my care is fourteen days; but this is made so high by ten of unusually long duration and great initial severity. In one very interesting example the duration was due to artificial lime-juice having been fraudulently substituted for pure by the druggist, and being used until I discovered the fraud by the impotency of the medicine: while at the Liverpool Northern Hospital, four days generally sufficed for convalescenee; and during seven years, only one case lasted for three weeks. Successive junior house-surgeons, fresh from the London Hospitals, as they arrived went through an interesting course of sneers doubts and confidence.

On being elected to the Liverpool Royal Infirmary, however, the plan met no such conspicuous success, and from the region of confidence I was myself beaten back into the domains of doubt.

Thus stood the point: London men, after a trial of the virtue of lime-juice, gave a verdict of not proven. The physicians at the Liverpool Royal Infirmary, at the very time when I, at the Northern Hospital, was meeting with a success which surprised myself, gave the medicine an ample trial' and abandoned it as unsatisfactory; and when I was transferred to the same institution, my own experience tallied with theirs. While at the one place I saw case after case so bad one day that all motion was impossible and the patients crying with the intensity of their sufferings, and three days afterwards walking about the wards apparently quite well; and this occurred so frequently that a duration of a fortnight in the hospital was an extraordinary occurrence. The sequence of cause and effect seemed as marked as anything could be. If the lime-juice was not used in a sufficient quantity, or was old, bad, or fictitious, there was no improvement; but as soon as the proper quantity and quality was secured, the restoration was immediate. I could as soon doubt the efficacy of opium in procuring sleep as I could the efficacy of lime-juice in curing acute rheumatism. Yet in another part of the town, in another institution, I began gradually to lose faith in the remedy. The reason of this I cannot as yet make out. It may be that there are varieties in disease of which we know little; that the complaint is influenced by local circumstances not yet thought of or understood. It may be, that, as some epidemics of small-pox are more deadly than others, so at one time the cases of rheumatic fever are mild, at others severe. It may be that epidemic influences vary in their intensity, just as malaria does; few now venture to deny the value of quinine in ague, yet every physician can recall instances in which it has been apparently useless.

To demonstrate, if possible, the cause of this uncertainty, I have treated my patients in a variety of ways. Having heard extraordinary vaunts of the value of large doses of carbonate of potash in one of the London Hospitals, I determined to test the plan fully. The result was a complete failure, and I was forced to the conclusion either that certain symptoms go by the name of acute rheumatism, which have no real claim to the title, or that experience gained in one locality is useless for another. As the doses used were in some cases sufficiently large to induce severe purging, there could be no doubt that the failure was not attributable to a feeble use of the drug.

After the carbonate of potash, I gave a full trial to the nitrate, after that to quinine, to opium, to wine, to steel, to cod-liver oil.

Nor did we omit the use of such simple remedies as liquor ammoniæ acetatis, and the still more simple one of pure water.

From none of these plans have I been able to obtain so satisfactory a result as from the treatment by lime-juice alone, although the balance in its favor over warmth, comfort, and nutritious diet, without medicine superadded, is not unvaryingly large.

The practical effect of the doubt, therefore, respecting lime-juice is simply to modify the belief in the constancy and certainty and celerity of its opersaion.

Of its superiority over any other medicine yet administered, I have no misgiving.

The way I employ it is simple; the patient is directed to take at least eight ounces of it in the day, and no other medicament of any kind whatever is used, unless it be opium to procure sleep at night. If the skin is very white, the tongue much loaded, and the perspiration excessive, two drachms of the tincture of sesquichloride of iron is given in addition during the twenty-four hours, and some wine at dinner-time and in the eveing. If, during the progress of the case, the hands or feet become unusually swelled or painful, they are merely wrapped up in cotton wool which has been freely sprinkled over with tincture of camphor.

If the heart become affected I make no difference in the plan proposed; I continue the lime-juice as if nothing unusual had occurred, with the full confidence that the complication will be evanescent, nor have I yet been deceived. When this accident occurs, mercury, bleeding, or cupping, seem to me to have the effect of aggravating the mischief and of rendering a transient complaint a permanent disease.

Of the *modus operandi* of lime-juice I can form no idea. Vegetable acids, *e, g.*, citric or tartaric, are not substitutes for it. Lemon-juice is inferior to it, though to a very small degree, so that we infer that it is not the particular acid which does the good. I have never known it purge, though it has seemed to gripe occasionally. It acts quietly yet almost certainly, as does arsenic in lepra, quina in ague, and colchicum in gout.

This treatment is very simple, and to the patient very pleasant. One of its chief advantages is, that it does not aggravate the extreme debility which attends and follows the fever.

It ought entirely to supersede the system of drenching, and so commonly practiced under the notion that a poison had to be eliminated out of, or destroyed in, the system.

I must add, that since the preceding pages were written, I have had under my care three unusually protracted cases of acute rheumatism, attended with extreme debility, total anorexia, and a constant tendency to relapse. These gave me opportunity of testing the real value of every suggestion hitherto offered, and all drugs proved equally worthless for cure; this was at last effected, and suddenly, by simply change of air.

These cases seemed to unsettle the conclusion already drawn; to a certain extent they do, but that extent is small. We do not lose faith in arsenic because many a case of lepra is uncured by it; nor do we have less confidence in quina because we see at times a man who is cinchonized have an ague fit; we still believe in mercury, though it often fails to remove "secondaries," and we still prescribe opium for the relief of pain, though it is powerless to arrest the agonies of gout.

In fine, we constantly have to confess that we possess no single panacea; that disease often baffles our best endeavors, and that the most skillful physician is but a man after all.

But though a man he may be a destroyer rather than a helper, and surely it is something to know how he may certainly escape being the former if we cannot invariably be the latter.

[Lime-juice may be procured in Liverpool at a shilling the quart bottle. It is generally taken out by vessels going long voyages. It is far more palatable than lemon-juice.]—*London Med. Review, March,* 1863, *p.* 458. *Braithwaite's Retrospect.*

